# A Novel Ferroptosis-Related Prognostic Signature Reveals Macrophage Infiltration and EMT Status in Bladder Cancer

**DOI:** 10.3389/fcell.2021.712230

**Published:** 2021-08-20

**Authors:** Yilin Yan, Jinming Cai, Zhengnan Huang, Xiangqian Cao, Pengfei Tang, Zeyi Wang, Fang Zhang, Shujie Xia, Bing Shen

**Affiliations:** ^1^Department of Urology, Shanghai General Hospital, Shanghai Jiao Tong University School of Medicine, Shanghai, China; ^2^Department of Urology, Shanghai General Hospital Affiliated to Nanjing Medical University, Shanghai, China; ^3^Institute of Urology, Shanghai Jiao Tong University, Shanghai, China

**Keywords:** bladder cancer, ferroptosis, prognostic signature, macrophage, EMT status

## Abstract

Bladder cancer (BC) belongs to one of the most common and highly heterogeneous malignancies. Ferroptosis is a newly discovered regulated cell death (RCD), characterized by accumulation of toxic lipid peroxides, and plays a crucial role in tumor progression. Here, we conducted a comprehensive analysis on the transcriptomics data of ferroptosis-related genes in BC based on The Cancer Genome Atlas (TCGA) and three Gene Expression Omnibus (GEO) datasets. In our study, a 6-gene signature was identified based on the potential prognostic ferroptotic regulatory genes. Furthermore, our signature revealed a good independent prognostic ability in BC. Patients with low-risk score exhibited higher FGFR3 mutation rates while high risk score had a positive association with higher RB1 mutation rates. Meanwhile, higher proportions of macrophages were observed in high BC risk group simultaneously with four methods. Unexpectedly, the risk score showed a significant positive correlation with epithelial-mesenchymal transition (EMT) status. Functional assays indicated that CRYAB and SQLE knockdown was associated with attenuated invasion capacity. Our study revealed a ferroptosis-related risk model for predicting prognostic and BC progression. Our results indicate that targeting ferroptosis may be a therapeutic strategy for BC.

## Introduction

Bladder cancer (BC) belongs to one of most common malignancies in genitourinary system which can been further classified into two subtypes: muscle-invasive bladder cancer (MIBC) and non-muscle-invasive bladder cancer (NMIBC) (Siegel et al., 2020). Conventional treatment options for BC include surgery, radiation, and cisplatin-based chemotherapy. Yet, despite considerable advances in diagnosis and treatment, BC still exhibits high rates of recurrence and metastasis due to the high-level heterogeneity and genomic instability of BC (Alfred Witjes et al., 2017; Dy et al., 2017). BC responds differently to treatment resulting from different driver events. For example, BC patients with a mesenchymal related signature appears resistant to platinum-based chemotherapy and sensitive to immunotherapy (Wang et al., 2018a). Therefore, there is an urgent need for the development of novel therapeutic strategies so as to improve outcome.

Conventional therapy aims to suppress tumor cells through activating a certain kind of regulated cell death (RCD) (Stockwell et al., 2017). Ferroptosis is a unique cell death pathway driven by iron-dependent lipid peroxidation. Increasing evidence has determined the pivotal role of ferroptotic regulatory genes, such as TP53 (Xie et al., 2017), CISD1 (Yuan et al., 2016), GPX4 (Ingold et al., 2018), FINO2 (Gaschler et al., 2018), in tumor progression of numerous cancer types. In addition, chemotherapeutic drugs can improve efficacy on various tumor cells when combined with ferroptosis inducer (Guo et al., 2018; Belavgeni et al., 2019). Notably, previous studies indicated the significant role of ferroptosis in diagnosis and treatment management of BC (Drayton et al., 2014; Guo et al., 2020). Therefore, ferroptosis can serve as a potential target for intervention in BC patients. However, there are still few scientific studies on the correlation between BC and ferroptosis. Despite significant progress in BC, few have considered the use of ferroptosis-related gene characteristics to construct a prognostic signature in BC. The precise underlying molecular mechanism and critical molecules of ferroptosis in BC progression remain to be illuminated.

Epithelial–mesenchymal transition (EMT), a plastic process in which epithelial cells gain mesenchymal characteristics, plays an important role in embryonic development (Sha et al., 2020; Yang et al., 2020a). Growing evidences showed that this physiological process was closely related to enhanced capacity for BC invasion (Schulz et al., 2019). Activation of EMT was reported to promote cell growth and metastasis in the BC (Wang et al., 2020). And we also proved this assumption in our previous research (Yan et al., 2020b). Moreover, evidence has shown that EMT-related gene and immune cell infiltration could impact outcomes in BC patients treated with immunotherapy. Infiltrated immune cells play important roles in iron homeostasis and ferroptosis (Wang et al., 2018b). Many immune cells, such as Th1 cells and macrophages, are reported to be involved in the maintenance of iron metabolism (Ganz and Nemeth, 2015). In addition, immunoregulation was found to efficiently inhibit tumor progression by synergistic ferroptosis. Reports claimed that increased ferroptotic level enhances the anti-tumor therapeutic effect of immunotherapy. Meanwhile, immunotherapy can activate T cells infiltration to promote the lipid-ROS formation and ferroptosis in tumor cells (Wang et al., 2019).

Herein, we evaluated publicly available BC datasets and identified differentially expressed ferroptosis-related genes strongly correlated to the prognosis of BC. Then we constructed a predictive risk model and validated its prognostic accuracy. Alterations of mutation profile and immune cell infiltration were also explored. Furthermore, functional enrichment analysis and the underlying mechanisms were ultimately confirmed via *in vitro* experiments. Our findings may help lead to a deeper understanding of BC progression and further provide novel therapeutic targets for BC.

## Materials and Methods

### Data Acquisition and Processing

All datasets used in this study were available to the public. Data of gene expression and clinical information were obtained from the Cancer Genome Atlas (TCGA) data portal,^1^ the GTEx database,^2^ GSE13507 dataset, GSE31684 dataset, GSE48075 datasets. Data from the GTEx database were selected to expand the subset of data from TCGA data portal. Then, Robust Multiarray Average was used to normalize the raw expression data (Irizarry et al., 2003). Sixty ferroptosis-related genes were obtained from the previous literature (Stockwell et al., 2017; Bersuker et al., 2019; Doll et al., 2019; Hassannia et al., 2019). HPA^3^ is a platform that contains representative immunohistochemistry images expression data for common kinds of cancers (Thul et al., 2017). In this study, images of protein expression of SQLE and CRYAB between normal and BC samples were directly visualized by HPA.

### Construction and Validation of the Prognostic Model

Differentially expressed genes (DEGs) between normal and tumor tissues were identified with the “limma” R package with a false discovery rate (FDR) <0.05 as cutoff threshold (Ritchie et al., 2015). BC patients in TCGA database were randomized into two groups at a ratio of 3:1 using the R package “caret.” Univariate Cox regression analysis of overall survival (OS) was used to identify prognostic ferroptosis-related genes by “survival” R package filtered by *p* < 0.05. Then, “Venn” R package was implemented to get the intersect genes between ferroptosis-related DEGs and prognostic genes. For the selection of predictor variables, Least Absolute Shrinkage and Selection Operator (LASSO) regression analysis was applied to construct a prognostic model using “glmnet” on R (Simon et al., 2011). At last, a ferroptosis-related prognostic model was identified by extracting the coefficients, and the risk score for each case was determined by multiplying the level of each selected gene with its corresponding coefficient. The BC tissues were then divided into low- and high-risk group according to the median risk score. Univariate and multivariate Cox regression analyses were utilized to explore whether the risk score calculated from our model could play as an independent prognostic factor for BC patients after considering other clinical factors including gender, age, stage, T and N stage. The results were acquired by application of the “forestplot” package. To assess the predictive power of the signature, survival analysis and area under the curve (AUC) was calculated with “timeROC” R package. Somatic mutation data, which stored in the form of Mutation Annotation Format (MAF), were visualized and analyzed using “maftools.” For subgroup analysis, patients were divided into different groups based on features as follows: gender (Male or Female), age (≤70 years old or >70 years old), stage status (Stage I, II or III, IV) and N status (N0 or >N0).

### Pathway Enrichment Analysis

To understand the underlying mechanisms affecting the risk signature, BC samples were divided into two cohorts depending on risk score of each patient, and then gene set enrichment analysis (GSEA) enrichment analysis was performed via “javaGSEA” to show the result (Subramanian et al., 2005). Nominal *p* < 0.05 and FDR < 0.05 were considered significant. Enrichment of hallmark pathways within modules on a single sample to calculate a signaling pathway variation score was implemented through gene set variation analysis via “gsva” R package (Hanzelmann et al., 2013).

### Evaluation of Immune Cell Infiltration

Transcriptome profiles from TCGA-BLCA cohort were used to estimate tumor immune infiltrate populations by TIMER, quanTIseq, TIP, and ssGSEA algorithm. TIMER^4^ is a method that it only makes estimations of six immune cell types, taking the tissue specificity into consideration (Li et al., 2020). QuanTIseq performs an absolute quantification of cell types in the samples (Finotello et al., 2019). Tumor ImmunoPhenotype (TIP) pipeline was applied for immune activity estimation (Xu et al., 2018). Single sample Gene Set Enrichment analysis (ssGSEA) was applied to evaluate the enrichment scores.

### Construction and Evaluation of the Nomogram

The clinical features and risk signature were extracted to construct a prognostic nomogram to assess the OS probability as a single numerical value. The “rms” R package was used to draw the nomogram (Feng et al., 2017). In addition, Calibration plots were generated to explore the performance characteristics of the nomograms. The clinical usefulness of the risk model was evaluated using decision curve analysis (DCA).

### Transient Transfections

For siRNA knockdown experiments, human BC cell lines were either transfected with CRYAB siRNA, SQLE siRNA, or non-silencing control siRNA, using Lipofectamine^®^ 3000 Reagent according to the manufacturer’s protocol. The sequences were as follows: siNC (non-coding control): 5′-UUCUCCGAACGUGUCACGU-3′; siSQLE: 5′-GGUGUUGUGUUACAGUUAU-3′; siCRYAB: 5′-CAGAGG AACUCAAAGUUAA-3′.

### RNA Extraction and RT-qPCR

Total RNA isolation and purification were performed using TRIzol RNA and then reverse-transcribed by Prime-Script Reverse Kit (TaKaRa) per manufacturer′s instructions. The expression levels of genes were quantified with SYBR Green. The primers used were the following: SQLE forward 5′-TGACAATTCTCATCTGAGGTCCA-3′, reverse 5′-CAGGGATACCCTTTAGCAGTTTT-3′; CRYAB forward 5′-CCTGAGTCCCTTCTACCTTCG-3′, reverse 5′-CACATCTCCCAACACCTTAACTT-3′.

### Western Blotting

Western Blotting was performed using the routine methods as described before (Yan et al., 2020b). Briefly, cells were lysed by RIPA buffer, then 20 μg proteins were separated by SDS-PAGE and transferred onto PVDF membrane. After blocking with 5% non-fat milk in PBST, the membrane was incubated with primary antibodies overnight at 4°C. β-actin was used as a loading control. The membranes were then incubated with secondary anti-body respectively. The Western blots were visualized using the ECL substrate kit. The antibodies against CRYAB, SQLE were purchased from Proteintech (Rosemont, IL, United States). β-actin were purchased from Sigma-Aldrich (Saint Louis, MO, United States).

### Transwell Invasion Assay

8.0-μm-pore polycarbonate membrane were used to assess cell invasion ability of BC cells as previously described (Yan et al., 2020b). Briefly, after the Matrigel was coagulated, 1 × 10^5^ cells were plated on the Matrigel. After incubation, cells were fixed in 4% formaldehyde and stained with crystal violet. Cells were visualized using light microscopy.

## Results

### Identification of Bladder Cancer-Specific Ferroptosis Related Genes and Construction of the Prognostic Model

**FIGURE 1:**
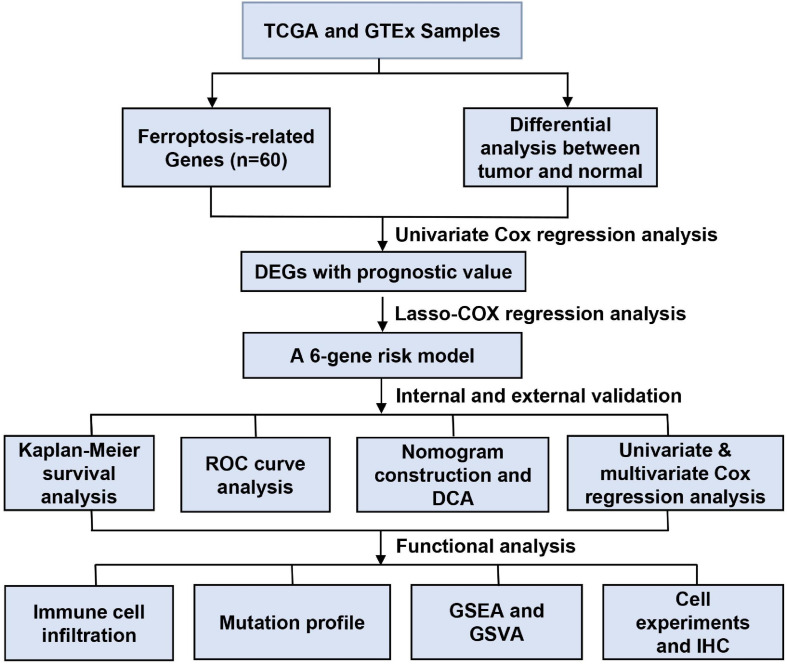
Data processing flow chart.

**FIGURE 2:**
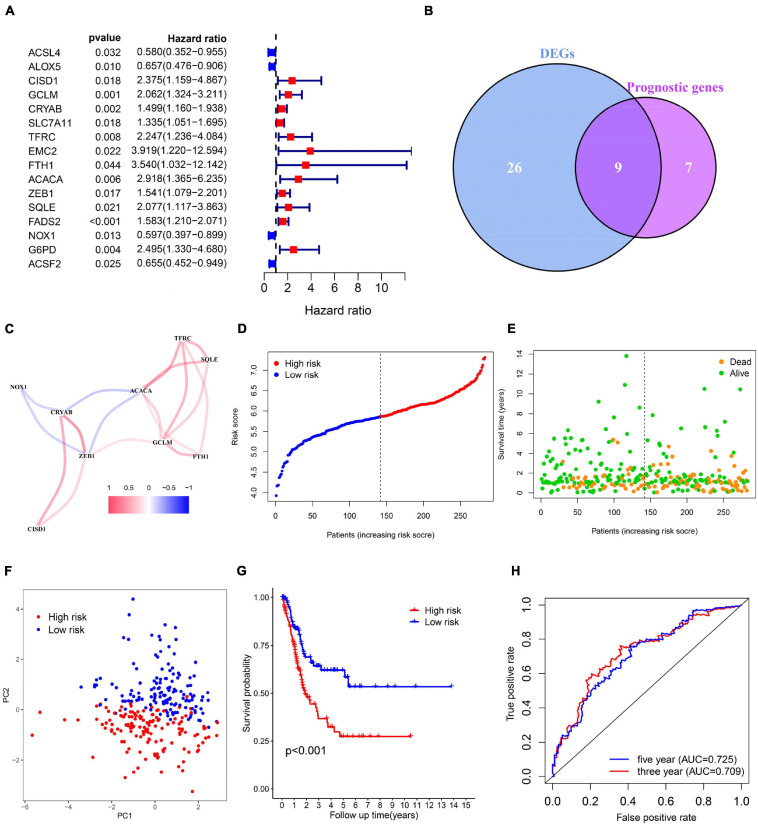
Identification of bladder cancer-specific ferroptosis-related genes and construction of the prognostic model. **(A)** Forest plots based on univariate survival analysis in patients with BC. **(B)** Venn diagram for overlapping genes among differentially expressed genes (DEG) correlated with patient outcome. **(C)** The correlation network of candidate genes. **(D–F)** Risk score distribution, overall survival (OS) status and PCA plot of high and low risk BC cases in the TCGA data. **(G)** Kaplan-Meier survival curves of patients in BC groups of different risk score in the TCGA cohort. **(H)** Time-dependent ROC curves of overall survival at 3- (red) and 5-years (blue).

Figure 1 shows the study flow chart. A total of 403 BC patients from the TCGA-BLCA, 165 patients from the GSE13507, 93 patients from the GSE31684 and 73 patients from the GSE 48075 were finally included. After data collection, 60 ferroptosis-related genes were identified. DEGs between normal and tumor tissues within these genes were identified and were subsequently analyzed by Cox regression and LASSO regression to construct a prognostic ferroptosis-related risk signature. This signature was further evaluated using Cox regression analysis, ROC curve analysis, clinical stratification analysis, mutation correlation analysis, immune cell infiltration comparison, and external dataset validation. Finally, an optimized model and nomogram were established. The three GEO datasets were subjected to validation of all results. To determine the prognostic value of the above ferroptosis-related genes in BC, univariate Cox regression analysis identified 16 genes as significantly correlated with BC survival (Figure 2A), and 9 of them were differentially expressed between tumor tissues and adjacent carcinoma tissues (Figure 2B). Expression correlations between these genes is shown in Figure 2C. LASSO and multivariable Cox regression analysis were performed to identify a risk model based on the expression level of the above nine genes (Supplementary Figure 1). A 6-gene signature was identified and risk score was calculated by following formula: (0.251 × CISD1) + (0.135 × CRYAB) + (0.225 × FTH1) + (0.656 × ACACA) + (0.242 × ZEB1) + (0.202 × SQLE).

Next, risk score of each BC tissue was calculated and patients were assigned into a low- or a high-risk group according to the median cut-off value (Figure 2D). Survival status of the BC patients was visualized in Figure 2E. The proportion of mortality in high-risk group was 48%, much higher than 27% in the low-risk group. PCA analysis showed clear separation of patients in different risk groups (Figure 2F). Kaplan-Meier survival curve revealed significant difference in OS between the groups. As demonstrated, more deaths happened in the high-risk groups than those in low-risk groups, suggesting that the risk model could accurately distinguish BC patients with poor prognosis. We then evaluated the predictive power and accuracy of the risk signature with ROC curve analysis. A time-dependent ROC curve was performed and the AUC was 0.709 at 3-years and 0.725 at 5-years (Figures 2G,H).

### Validation of the 6-Gene Risk Model

**FIGURE 3:**
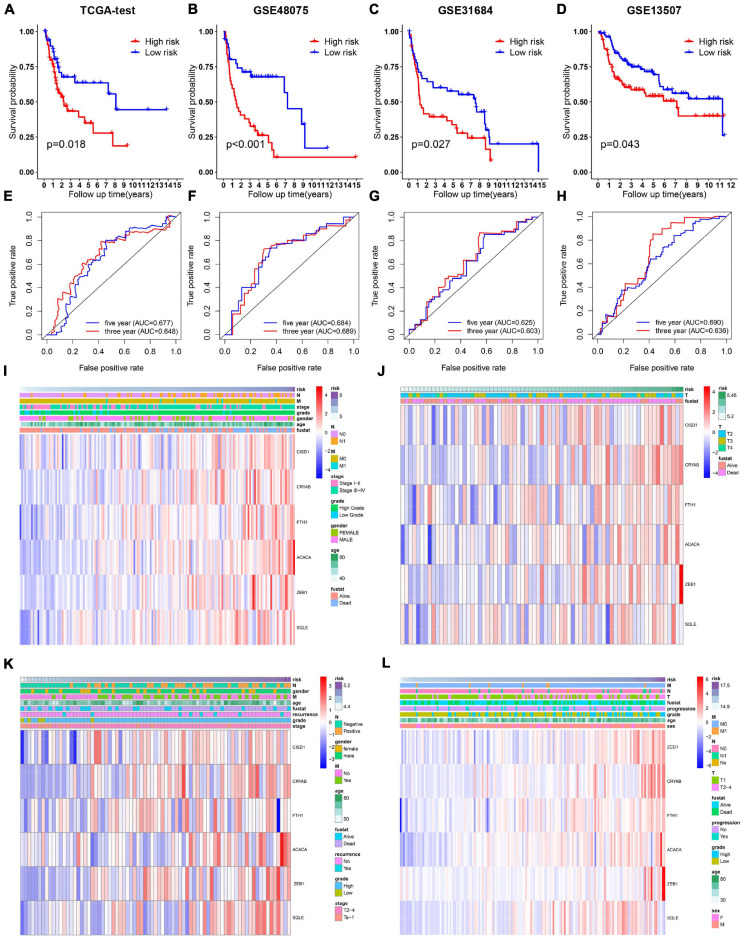
Validation of the 6-gene signature model in the internal TCGA and another indicated three independent public datasets. **(A–D)** Kaplan-Meier survival curves for bladder cancer patients based on risk score in TCGA, GSE48075, and GSE31684, and GSE13507 datasets. **(E–H)** ROC curve analysis for predicting 3- and 5-year overall survival in the above four BC cohorts. **(I–L)** Heatmaps showing the expression level of 6 selected ferroptosis-related genes ranked by risk score in the above four BC cohorts.

To evaluate whether the risk model exhibits similar predictive performance and accuracy in other BC patient cohorts, robustness of the 6-gene signature was then tested in TCGA internal cohort and another three independent GEO BC cohorts. Survival analysis results confirmed that patients of high-risk correlated with poor OS (Figures 3A–D). As shown in Figures 3E–H, the AUC was 0.677, 0.684, 0.625, 0.690 for 5-year survival prediction and 0.648, 0.689, 0.603, 0.636 for 3-year survival prediction in TCGA, GSE48075, GSE31684, and GSE13507. Heat maps were shown to present the expression levels of the six genes and clinical features ordered by risk score in the above four datasets. Clinical and pathological features, such as TNM stage and grade were enriched BC cases with high-risk score (Figures 3I–L). Therefore, we believe that the ferroptosis-based risk model indicates good prediction stability and performance.

### Prognosis Analysis of the 6-Gene Risk Signature With Clinicopathological Features

**FIGURE 4:**
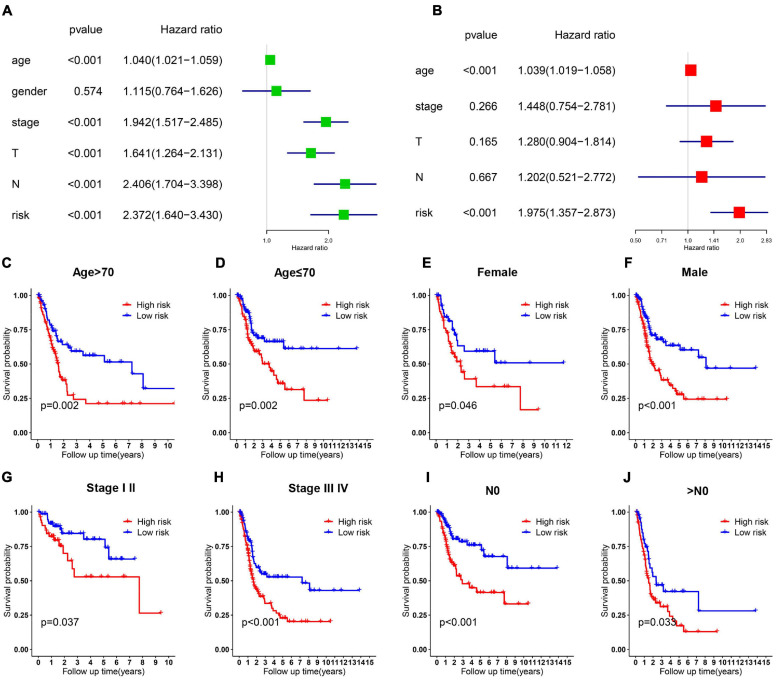
Cox regression and stratified analysis of the 6 gene risk model. **(A,B)** Results of univariate and multivariate regression analysis of the relation between clinical features and risk model. **(C–J)** Kaplan–Meier survival curves for bladder cancer patients with age **(C,D)**, gender **(E,F)**, stage **(G,H)**, and N metastasis **(I,J)**.

Relationship between OS, clinicopathological parameters and risk score were further analyzed to assess whether the risk score was an independent predictor of outcome. Univariate and multivariate Cox analysis were used based on OS of BC patients, using the co-variables including risk score, age, gender, stage, T and N stage to validate the independence of the risk model among other clinic-pathologic characteristics. Results from univariate and corresponding multivariable Cox regression analysis demonstrated that our risk model could serve as an independent prognostic factor (*p* < 0.05; Figures 4A, B). Then we further evaluated the effects of clinical characteristics, such as different stage and lymph node metastatic status on outcome among BC patients, and found that high risk score was highly correlated with poor outcome (Figures 4C–J). These results demonstrated that the ferroptosis-related prognostic model could predict the prognosis of patients with BC regardless of clinical conditions.

### Construction and Evaluation of a Predictive Nomogram

**FIGURE 5:**
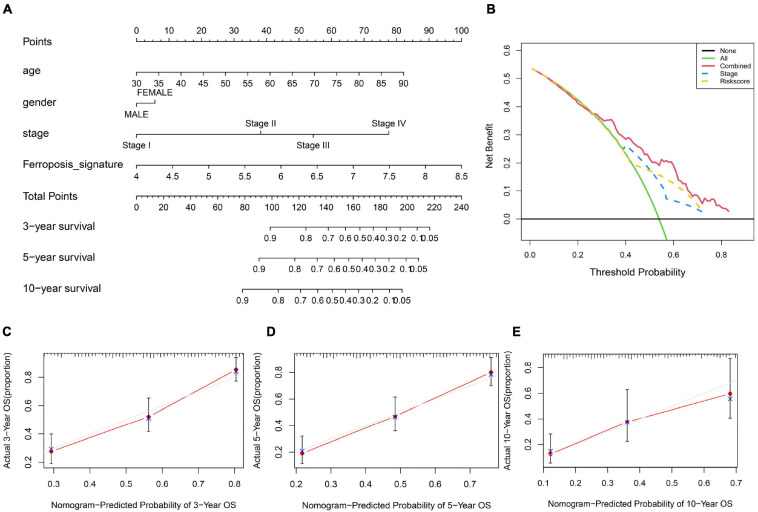
Construction and validation of a nomogram. **(A)** Prognostic nomogram predicting overall survival probability of BC patients. **(B)** DCA curves analysis for the nomogram. **(C–E)** Calibration curves of the nomogram for predicting overall survival probability.

Furthermore, we utilized a quantitative method by integrating the risk score and clinical features to construct a nomogram (Figure 5A). Female patients had a higher risk of a poor prognosis while the higher age, tumor stage and risk score indicated a lower survival rate in patients. Combined with our risk model and clinical feature, net benefits were presented in our DCA curve, and we found that compared to a single factor, the combined showed the optimal net benefit (Figure 5B). Meanwhile, the calibration plot for the probability of 3-, 5-, and 10-year survival showed an optimal consistency between observation and predictive curves (Figures 5C–E). These results indicate that the nomogram has proper clinical applicability.

### Correlation of Mutation and Immune Cell Infiltration Landscape With the Risk Model

**FIGURE 6:**
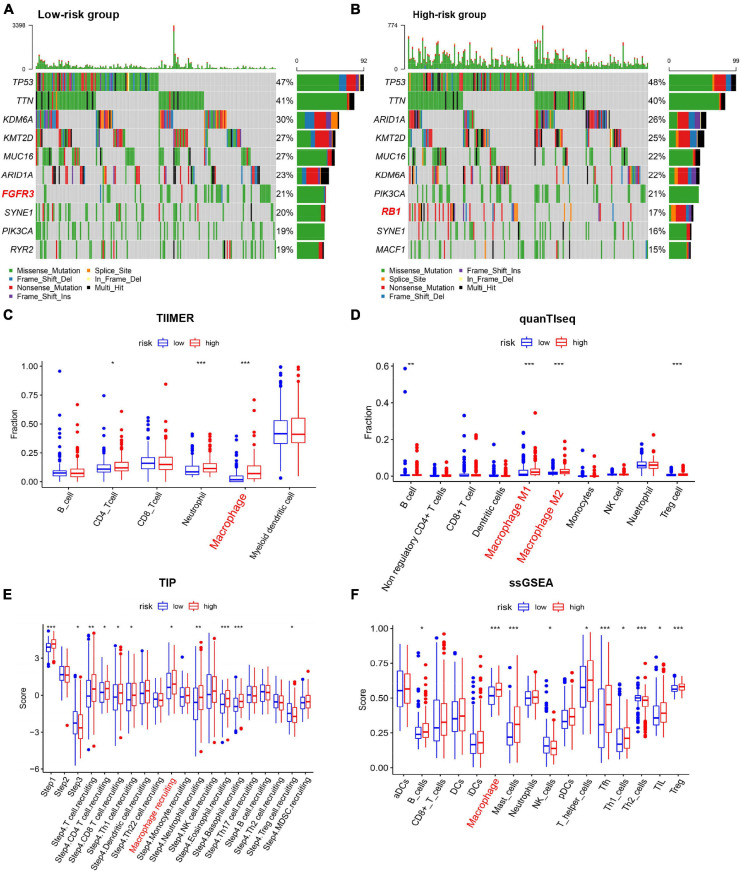
Characterization of immune cell infiltration and mutation landscape in BC tissues among different risk groups. **(A,B)** Somatic mutation profiles of BC tissues among different risk groups. **(C–F)** Boxplots of Immune cell infiltration with high and low risk scores estimated using TIMER, quanTIseq, TIP, and ssGSEA. *p < 0.05, **p < 0.01, ***p < 0.001.

To determine whether the risk model was correlated with tumor mutation of BC, relationship between the model and gene mutations was analyzed. The profile of somatic mutation is visualized in Supplementary Figure 2. The mutation profile distribution based on patients’ risk score were shown in Figures 6A,B. Notably, the low-risk tumor groups have significantly lower rates of FGFR3 mutation while the overall mutation rates of RB1 appeared to be higher in the high-risk subgroup. FGFR mutation was reported to be significantly enriched in the luminal papillary subtype, characterized by lower stage, while high RB1 mutation frequencies are significantly high in basal-squamous and neuronal subtypes, indicating lower survival rate. Then we evaluated the TIICs proportions with the aid of TIMER, quanTIseq, TIP and ssGSEA, four well-accepted methods for comprehensive analysis of TIICs. Two distinct patterns of immune infiltrate were observed in high and low-risk cohorts. Surprisingly, tissues with high risk generally had higher density of macrophage (Figures 6C–F). Increased proportions of tumor-infiltrating macrophages have been reported to be involved in tumor progression and metastasis of BC. These evidences demonstrated that high RB1 mutation and tumor-infiltrating macrophages may account for the poor prognosis in high-risk group.

### Identification of the 6-Gene Risk Model Correlated Biological Pathways

**FIGURE 7:**
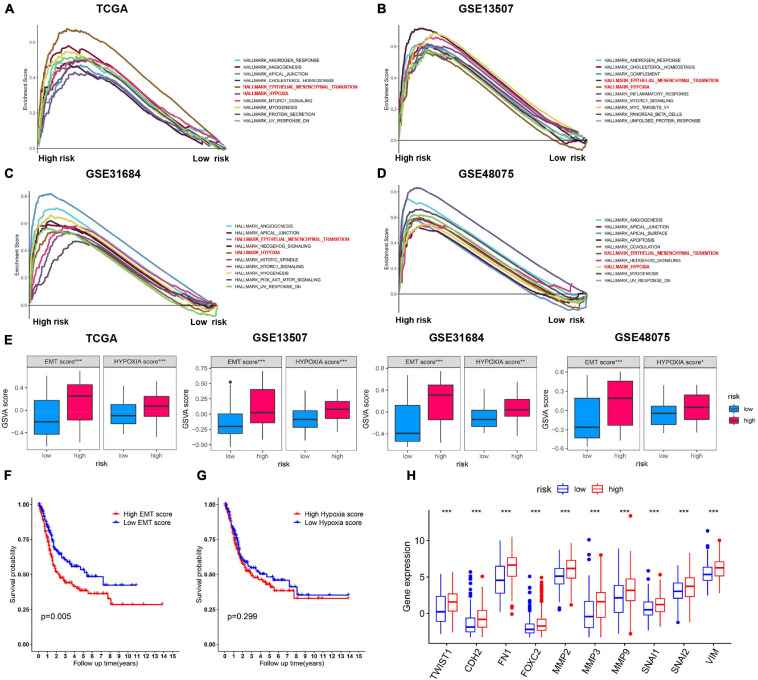
Validation of the expression patterns of risk model. **(A–D)** Top 10 enriched hallmark gene sets with high and low risk score in TCGA, GSE13507, GSE48075, and GSE31684 datasets. **(E)** GSVA score of EMT and hypoxia gene set in the above four BC cohorts. **(F,G)** Kaplan–Meier survival curves for BC patients classified by EMT and hypoxia score. **(H)** The correlation analysis between the expression levels for EMT-related genes and the risk subgroups. ***p < 0.001.

Gene set enrichment analysis (GSEA) analysis of BC patients with a different risk score was performed to investigate the possible biological function of the risk model in the carcinogenesis of BC. The results showed that, in four BC groups, high-risk score was positively correlated to important tumor related pathways, especially “HALLMARK_EMT” and “HALLMARK_HYPOXIA” (Figures 7A–D). Meanwhile, the GSVA scores of such two pathways were significantly higher in high-risk score cohort, respectively (Figure 7E). Survival analysis showed a positive correlation between EMT score and the OS in patients with BC (Figure 7F), while the difference was not significant for HYPOXIA score (Figure 7G). In addition, expression of EMT-related markers was elevated in high-risk group (Figure 7H). These results indicated that the risk model played a critical role in predicting EMT-related function in BC.

### Functions of the Identified Biomarkers in BC Invasion

**FIGURE 8:**
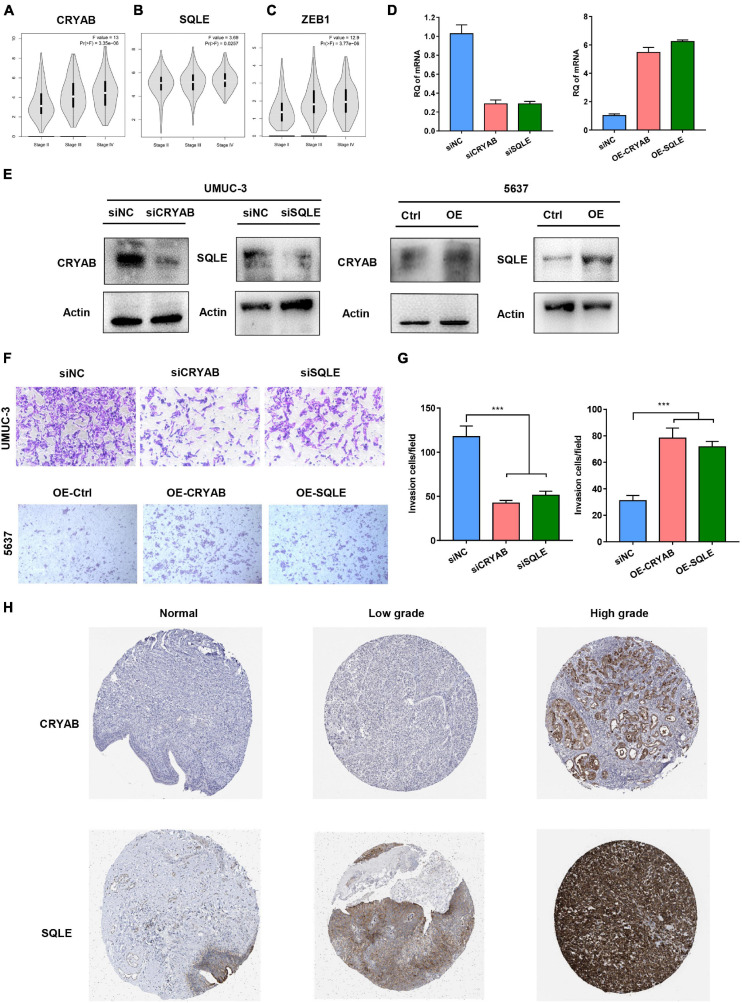
Functions of the Identified Biomarkers in BC Invasion. **(A–C)** The correlation between expression of CRYAB, SQLE, ZEB1, and the clinical stage in different BC stages. **(D,E)** Knockdown and overexpression efficiency was confirmed by real-time RT-PCR and Western blot. **(F,G)** Transwell invasion assay analyzed the effect of CRYAB and SQLE knockdown and overexpression on cell invasion *in vitro*. Scale bar, 100 μm. **(H)** Immunohistochemical (IHC) staining of the expression of CRYAB and SQLE in normal urothelium and BC tissues with different degree of malignancy. ***p < 0.001.

EMT progress was previously reported to contribute to cancer cell invasion, resulting in rapid tumor development. Within the six genes, higher level of CRYAB, SQLE, and ZEB1 significantly correlated with advanced stage (Figures 8A–C). Considering the important role of ZEB1 in tumor metastasis, we thus focused on another two genes. Then we performed functional studies after transfection using specific small interfering RNAs (siRNAs) (Figures 8D, E). The results showed that knockdown of CRYAB and SQLE in BC cell lines inhibited cell migration and invasion ability (Figures 8F, G). Moreover, overexpression of these two genes promoted the invasive ability of bladder tumor cell (Figures 8D–G). Immunohistochemical analysis indicated the positive staining intensity for CRYAB and SQLE in BC tissues as significantly stronger than in the normal urothelium tissues. Moreover, BC tissues with high malignancy exhibited strong staining intensity than tissues with low malignancy by HPA (Figure 8H). These results confirmed that CRYAB and SQLE were correlated well with the grade of tumorigenesis.

## Discussion

Bladder cancer (BC) has been reported among the most common malignancies, presenting a major health burden for society. How to optimize therapeutic protocols for mortality reduction of patients with advanced BC remains a challenge. Therefore, there is an urgent need to identify key biomarkers that affect the outcome of BC. With future expansion of the database and multi-omics data, improved data mining algorithms can have an essential impact on tumor biology (Angus et al., 2019; Nacev et al., 2019; Liu et al., 2020; Tabassum et al., 2020). Transcriptome profiling provided us with novel insights into assessing prognostic of the individual patient when combining the corresponding clinical information. Multiple risk signatures have been identified in various tumors for the past few years (Yu et al., 2019; Yan et al., 2020a). However, most studies have failed to be applied to clinical practice owning to lack of systematic evaluation and a broad roll-out. Therefore, these questions illustrate the urgent need to identify the prognostic factors of BC to predict to identify high-risk populations.

Increasing evidence has shown that induction of cell death is among the most effective anticancer strategy and ferroptosis belongs to a RCD in which iron metabolism plays a vital role (Badgley et al., 2020; Yang et al., 2020b). However, molecular changes and mechanism of ferroptosis in BC has yet to be elucidated. In the current research, we have identified a ferroptosis-related risk model for BC. We used BC samples from TCGA as training group, GSE13507, GSE48075, and GSE31684 as validation groups. Our risk model is composed of6 differentially expressed ferroptosis-related genes (CISD1, CRYAB, FTH1, ACACA, ZEB1, SQLE) that correlate with patient outcome. We found that risk scores of our model were highly associated with stage and metastatic status. We further demonstrated that the risk model serves as an accurate predictor of BC survival and an independent predictor for BC prognosis by validating it in another three independent BC datasets. Besides, the nomogram performed well with a good calibration, indicating that the model is an accurate prognostic tool. Our results suggested that the model can well distinguish BC patients and predict prognosis, thereby helping to develop optimal treatment options based on risk score.

We identified several ferroptosis-related genes that predict the outcome of BC patients. Most of the genes have been reported in previous studies to be closely related to ferroptosis and cancer development of malignancies. CDGSH iron sulfur domain 1 (CISD1) is an iron-containing protein and could negatively modulates ferroptosis. Increasing stabilization of CISD1 inhibits erastin-induced mitochondrial iron uptake and oxidative damage (Yuan et al., 2016). Crystallin alpha B (CRYAB, also known as HSP beta-5) belongs to the small heat shock protein (HSP20) family and is reported to regulate iron uptake and GPX4 abundance (Stockwell et al., 2017). Ferritin heavy chain 1 (FTH1) is one of the subunits of Ferritin and plays an essential role in cellular iron balance in ferroptosis. Knockdown of FTH1 could lead to iron overabsorption and promote ferroptosis in the intestines of mice (Sun et al., 2016). acetyl-CoA carboxylase alpha (ACACA) is implicated in catalyzing the committed steps in fatty acids’ biosynthesis A and was reported to suppress FIN56-, but not erastin- or RSL3- induced ferroptosis (Shimada et al., 2016). Zinc finger E-box-binding homeobox 1 (ZEB1) function as a transcription factor that influences cell developmental and homeostasis cell fates. Knockout of ZEB1 suppressed GPX4-depletion-induced ferroptosis (Viswanathan et al., 2017). Squalene monooxygenase (SQLE) is a key rate-limiting enzyme in the biosynthesis of cholesterol. Overexpression of SQLE sensitizes ALCL cells to ferroptosis (Garcia-Bermudez et al., 2019).

Studies have indicated that gene mutations play an important role in the development of carcinoma including BC (Lawson et al., 2020). Mutations of the several genes, such as p53, FGFR3 and RB1, have been reported to be involved in BC with a high incidence (Robertson et al., 2018). Therefore, we further attempt to study whether there were gene mutation alterations between different groups in BC patients. Interestingly, in our study, higher FGFR3 mutation rate was observed in low-risk BC patients. FGFR mutation was significantly enriched in the luminal papillary subtype, characterized by lower stage, lower risk for progression and papillary morphology. These results suggested specific inhibitors designed against FGFR3 as a treatment option for these patients. Conversely, patients of high-risk score accounted for higher RB1 mutation. High RB1 mutation frequencies are significantly high in basal-squamous and neuronal subtypes, indicating worse OS and resistance to platinum-based chemotherapy. Immune cells were reported to be involved in the process of iron metabolism and ferroptosis level could promote the anti-tumor efficacy of immunotherapy (Nairz and Weiss, 2020; Tang et al., 2020). Interestingly, the infiltrating immune cells exhibit significant disparities among different groups in our study. Additionally, with four well-accepted methods, our signature identified higher proportion of macrophages in the high-risk group compared with the low-risk group. Previous researches have shown that increased proportions of tumor-infiltrating macrophages contributed to a propensity of metastases and poor prognosis for BC (Chen et al., 2018; Kobatake et al., 2020). Therefore, high infiltration of tumor-associated macrophages in patients with high risk may be an explanation for their poor outcome.

To investigate the underlying mechanisms, GSEA and GSVA were subsequently subjected to the pathway enrichment of each group. Interestingly, the enrichment analysis revealed that high risks were significantly enriched in EMT and hypoxia hallmark pathways. Of these two, only EMT was significantly associated with poor prognosis. EMT is an essential biological process, which plays a vital role in modulating tissue homeostasis and development (Bakir et al., 2020). Besides, EMT has been implicated in tumor progression and metastasis. In our study, high levels of EMT gene expression were observed in high-risk BC patients. Among genes of the risk model, we identified three genes (CRYAB, SQLE, and ZEB1) that were found to be was significantly and positively associated with clinical stage. Numerous studies have reported that ZEB1 contributes to cancer progression, while the role of CRYAB and SQLE in BC is rarely reported. We found that knockdown of CRYAB or SQLE significantly attenuated the invasive abilities of BC cells. Nevertheless, the potential mechanism of the two biomarkers might contribute to the carcinogenesis of BC remain unknown, and further investigation of potential mechanisms is needed.

Nonetheless, some limitations are inevitable. This risk model is highly dependent upon public databases. Further investigations need to be undertaken in future clinical researches. Moreover, protein level could differ from RNA expression, making it unavoidable to validate its clinical utility in more sets.

In summary, this research is the first to identify a novel signature of ferroptosis-related genes for predicting outcomes of BC patients. Results suggest that variation in gene mutation, immune response and EMT status might be several possible reasons for this model’s prognostic ability. These findings may help unveil new targets for the prevention, diagnosis, and treatment of BC.

## Data Availability Statement

The datasets presented in this study can be found in online repositories. The names of the repository/repositories and accession number(s) can be found in the article/Supplementary Material.

## Author Contributions

BS and SX: conception and design. YY and FZ: acquisition of the data. YY and JC: analysis of the data. YY, ZH, and XC: writing, review, and revision of the manuscript. PT and ZW: administrative, technical, or material support. BS: study supervision. All authors read and approved the final manuscript.

## Conflict of Interest

The authors declare that the research was conducted in the absence of any commercial or financial relationships that could be construed as a potential conflict of interest.

## Publisher’s Note

All claims expressed in this article are solely those of the authors and do not necessarily represent those of their affiliated organizations, or those of the publisher, the editors and the reviewers. Any product that may be evaluated in this article, or claim that may be made by its manufacturer, is not guaranteed or endorsed by the publisher.

## Footnotes

1
https://portal.gdc.cancer.gov/

2
https://xena.ucsc.edu/

3
http://www.proteinatlas.org

4
https://cistrome.shinyapps.io/timer/

## References

[B1] Alfred Witjes J. Lebret T. Comperat E. M. Cowan N. C. De Santis M. Bruins H. M. (2017). Updated 2016 EAU guidelines on muscle-invasive and metastatic bladder cancer. *Eur. Urol.* 71 462–475. 10.1016/j.eururo.2016.06.020 27375033

[B2] Angus L. Smid M. Wilting S. M. van Riet J. Van Hoeck A. Nguyen L. (2019). The genomic landscape of metastatic breast cancer highlights changes in mutation and signature frequencies. *Nat. Genet.* 51 1450–1458. 10.1038/s41588-019-0507-7 31570896 PMC6858873

[B3] Badgley M. A. Kremer D. M. Maurer H. C. DelGiorno K. E. Lee H. J. Purohit V. (2020). Cysteine depletion induces pancreatic tumor ferroptosis in mice. *Science* 368 85–89. 10.1126/science.aaw9872 32241947 PMC7681911

[B4] Bakir B. Chiarella A. M. Pitarresi J. R. Rustgi A. K. (2020). EMT. MET, Plasticity, and Tumor Metastasis. *Trends Cell Biol.* 30 764–776. 10.1016/j.tcb.2020.07.003 32800658 PMC7647095

[B5] Belavgeni A. Bornstein S. R. von Mässenhausen A. Tonnus W. Stumpf J. Meyer C. (2019). Exquisite sensitivity of adrenocortical carcinomas to induction of ferroptosis. *Proc. Natl. Acad. Sci. USA* 116 22269–22274. 10.1073/pnas.1912700116 31611400 PMC6825277

[B6] Bersuker K. Hendricks J. M. Li Z. Magtanong L. Ford B. Tang P. H. (2019). The CoQ oxidoreductase FSP1 acts parallel to GPX4 to inhibit ferroptosis. *Nature* 575 688–692. 10.1038/s41586-019-1705-2 31634900 PMC6883167

[B7] Chen C. He W. Huang J. Wang B. Li H. Cai Q. (2018). LNMAT1 promotes lymphatic metastasis of bladder cancer via CCL2 dependent macrophage recruitment. *Nat. Commun.* 9:3826. 10.1038/s41467-018-06152-x 30237493 PMC6148066

[B8] Doll S. Freitas F. P. Shah R. Aldrovandi M. da Silva M. C. Ingold I. (2019). FSP1 is a glutathione-independent ferroptosis suppressor. *Nature* 575 693–698. 10.1038/s41586-019-1707-0 31634899

[B9] Drayton R. M. Dudziec E. Peter S. Bertz S. Hartmann A. Bryant H. E. (2014). Reduced expression of miRNA-27a modulates cisplatin resistance in bladder cancer by targeting the cystine/glutamate exchanger SLC7A11. *Clin. Cancer Res.* 20 1990–2000. 10.1158/1078-0432.ccr-13-2805 24516043 PMC3974662

[B10] Dy G. W. Gore J. L. Forouzanfar M. H. Naghavi M. Fitzmaurice C. (2017). Global burden of urologic cancers, 1990-2013. *Eur. Urol.* 71 437–446. 10.1016/j.eururo.2016.10.008 28029399

[B11] Feng L. H. Dong H. Lau W. Y. Yu H. Zhu Y. Y. Zhao Y. (2017). Novel microvascular invasion-based prognostic nomograms to predict survival outcomes in patients after R0 resection for hepatocellular carcinoma. *J. Cancer Res. Clin. Oncol.* 143 293–303. 10.1007/s00432-016-2286-1 27743138 PMC11819416

[B12] Finotello F. Mayer C. Plattner C. Laschober G. Rieder D. Hackl H. (2019). Molecular and pharmacological modulators of the tumor immune contexture revealed by deconvolution of RNA-seq data. *Genome Med.* 11:34. 10.1186/s13073-019-0638-6 31126321 PMC6534875

[B13] Ganz T. Nemeth E. (2015). Iron homeostasis in host defence and inflammation. *Nat. Rev. Immunol.* 15 500–510. 10.1038/nri3863 26160612 PMC4801113

[B14] Garcia-Bermudez J. Baudrier L. Bayraktar E. C. Shen Y. La K. Guarecuco R. (2019). Squalene accumulation in cholesterol auxotrophic lymphomas prevents oxidative cell death. *Nature* 567 118–122. 10.1038/s41586-019-0945-5 30760928 PMC6405297

[B15] Gaschler M. M. Andia A. A. Liu H. Csuka J. M. Hurlocker B. Vaiana C. A. (2018). FINO(2) initiates ferroptosis through GPX4 inactivation and iron oxidation. *Nat. Chem. Biol.* 14 507–515. 10.1038/s41589-018-0031-6 29610484 PMC5899674

[B16] Guo J. Xu B. Han Q. Zhou H. Xia Y. Gong C. (2018). Ferroptosis: a novel anti-tumor action for cisplatin. *Cancer Res. Treat.* 50 445–460. 10.4143/crt.2016.572 28494534 PMC5912137

[B17] Guo P. Wang L. Shang W. Chen J. Chen Z. Xiong F. (2020). Intravesical in situ immunostimulatory gel for triple therapy of bladder cancer. *ACS Appl. Mater. Interfaces* 12 54367–54377. 10.1021/acsami.0c15176 33236624

[B18] Hassannia B. Vandenabeele P. Vanden Berghe T. (2019). Targeting ferroptosis to iron out cancer. *Cancer Cell* 35 830–849. 10.1016/j.ccell.2019.04.002 31105042

[B19] Hanzelmann S. Castelo R. Guinney J. (2013). GSVA: gene set variation analysis for microarray and RNA-seq data. *BMC Bioinform.* 14:7. 10.1186/1471-2105-14-7 23323831 PMC3618321

[B20] Ingold I. Berndt C. Schmitt S. Doll S. Poschmann G. Buday K. (2018). Selenium utilization by gpx4 is required to prevent hydroperoxide-induced ferroptosis. *Cell* 172 409–422. 10.1016/j.cell.2017.11.048 29290465

[B21] Irizarry R. A. Hobbs B. Collin F. Beazer-Barclay Y. D. Antonellis K. J. Scherf U. (2003). Exploration, normalization, and summaries of high density oligonucleotide array probe level data. *Biostatistics* 4 249–264. 10.1093/biostatistics/4.2.249 12925520

[B22] Kobatake K. Ikeda K. I. Nakata Y. Yamasaki N. Ueda T. Kanai A. (2020). Kdm6a deficiency activates inflammatory pathways, promotes m2 macrophage polarization, and causes bladder cancer in cooperation with p53 dysfunction. *Clin. Cancer Res.* 26 2065–2079. 10.1158/1078-0432.ccr-19-2230 32047002

[B23] Lawson A. R. J. Abascal F. Coorens T. H. H. Hooks Y. O’Neill L. Latimer C. (2020). Extensive heterogeneity in somatic mutation and selection in the human bladder. *Science* 370 75–82. 10.1126/science.aba8347 33004514

[B24] Li T. Fu J. Zeng Z. Cohen D. Li J. Chen Q. (2020). TIMER2.0 for analysis of tumor-infiltrating immune cells. *Nucleic Acids Res.* 48 W509–W514. 10.1093/nar/gkaa407 32442275 PMC7319575

[B25] Liu J. Wang Y. Chu Y. Xu R. Zhang D. Wang X. (2020). Identification of a TLR-induced four-lncRNA signature as a novel prognostic biomarker in esophageal carcinoma. *Front. Cell Dev. Biol.* 8:649. 10.3389/fcell.2020.00649 32850794 PMC7396588

[B26] Nacev B. A. Feng L. Bagert J. D. Lemiesz A. E. Gao J. Soshnev A. A. (2019). The expanding landscape of ‘oncohistone’ mutations in human cancers. *Nature* 567 473–478. 10.1038/s41586-019-1038-1 30894748 PMC6512987

[B27] Nairz M. Weiss G. (2020). Iron in infection and immunity. *Mol. Aspects Med.* 75:100864. 10.1016/j.mam.2020.100864 32461004

[B28] Ritchie M. E. Phipson B. Wu D. Hu Y. Law C. W. Shi W. (2015). limma powers differential expression analyses for RNA-sequencing and microarray studies. *Nucleic Acids Res.* 43:e47. 10.1093/nar/gkv007 25605792 PMC4402510

[B29] Robertson A. G. Kim J. Al-Ahmadie H. Bellmunt J. Guo G. Cherniack A. D. (2018). Comprehensive molecular characterization of muscle-invasive bladder cancer. *Cell* 174:1033. 10.1016/j.cell.2018.07.036 30096301 PMC6297116

[B30] Schulz A. Gorodetska I. Behrendt R. Fuessel S. Erdmann K. Foerster S. (2019). Linking NRP2 with EMT and chemoradioresistance in bladder cancer. *Front. Oncol.* 9:1461. 10.3389/fonc.2019.01461 32038994 PMC6986262

[B31] Sha Y. Wang S. Zhou P. Nie Q. (2020). Inference and multiscale model of epithelial-to-mesenchymal transition via single-cell transcriptomic data. *Nucleic Acids Res.* 48 9505–9520. 10.1093/nar/gkaa725 32870263 PMC7515733

[B32] Shimada K. Skouta R. Kaplan A. Yang W. S. Hayano M. Dixon S. J. (2016). Global survey of cell death mechanisms reveals metabolic regulation of ferroptosis. *Nat. Chem. Biol.* 12 497–503. 10.1038/nchembio.2079 27159577 PMC4920070

[B33] Siegel R. L. Miller K. D. Jemal A. (2020). Cancer statistics, 2020. *CA Cancer J. Clin.* 70 7–30. 10.3322/caac.21590 31912902

[B34] Simon N. Friedman J. Hastie T. Tibshirani R. (2011). Regularization paths for cox’s proportional hazards model via coordinate descent. *J. Stat. Softw.* 39 1–13. 10.18637/jss.v039.i05 27065756 PMC4824408

[B35] Stockwell B. R. Friedmann Angeli J. P. Bayir H. Bush A. I. Conrad M. Dixon S. J. (2017). Ferroptosis: a regulated cell death nexus linking metabolism. redox biology, and disease. *Cell* 171 273–285. 10.1016/j.cell.2017.09.021 28985560 PMC5685180

[B36] Subramanian A. Tamayo P. Mootha V. K. Mukherjee S. Ebert B. L. Gillette M. A. (2005). Gene set enrichment analysis: a knowledge-based approach for interpreting genome-wide expression profiles. *Proc. Natl. Acad. Sci. USA* 102 15545–15550. 10.1073/pnas.0506580102 16199517 PMC1239896

[B37] Sun X. Ou Z. Chen R. Niu X. Chen D. Kang R. (2016). Activation of the p62-Keap1-NRF2 pathway protects against ferroptosis in hepatocellular carcinoma cells. *Hepatology* 63 173–184. 10.1002/hep.28251 26403645 PMC4688087

[B38] Tabassum N. Cereser B. Stebbing J. (2020). A cell-cycle signature classifier for pan-cancer analysis. *Oncogene* 39 6041–6042. 10.1038/s41388-020-01426-5 32820254

[B39] Tang R. Xu J. Zhang B. Liu J. Liang C. Hua J. (2020). Ferroptosis, necroptosis, and pyroptosis in anticancer immunity. *J. Hematol. Oncol.* 13:110. 10.1186/s13045-020-00946-7 32778143 PMC7418434

[B40] Thul P. J. Åkesson L. Wiking M. Mahdessian D. Geladaki A. Ait Blal H. (2017). A subcellular map of the human proteome. *Science* 356:aal3321. 10.1126/science.aal3321 28495876

[B41] Viswanathan V. S. Ryan M. J. Dhruv H. D. Gill S. Eichhoff O. M. Seashore-Ludlow B. (2017). Dependency of a therapy-resistant state of cancer cells on a lipid peroxidase pathway. *Nature* 547 453–457. 10.1038/nature23007 28678785 PMC5667900

[B42] Wang L. Saci A. Szabo P. M. Chasalow S. D. Castillo-Martin M. Domingo-Domenech J. (2018a). EMT- and stroma-related gene expression and resistance to PD-1 blockade in urothelial cancer. *Nat. Commun.* 9:3503. 10.1038/s41467-018-05992-x 30158554 PMC6115401

[B43] Wang W. Green M. Choi J. E. Gijón M. Kennedy P. D. Johnson J. K. (2019). CD8(+) T cells regulate tumour ferroptosis during cancer immunotherapy. *Nature* 569 270–274. 10.1038/s41586-019-1170-y 31043744 PMC6533917

[B44] Wang Y. Yu L. Ding J. Chen Y. (2018b). Iron metabolism in cancer. *Int. J. Mol. Sci.* 20:95. 10.3390/ijms20010095 30591630 PMC6337236

[B45] Wang Z. Shang J. Li Z. Li H. Zhang C. He K. (2020). PIK3CA Is regulated by CUx1, promotes cell growth and metastasis in bladder cancer via activating epithelial-mesenchymal transition. *Front. Oncol.* 10:536072. 10.3389/fonc.2020.536072 33344221 PMC7744743

[B46] Xie Y. Zhu S. Song X. Sun X. Fan Y. Liu J. (2017). The tumor suppressor p53 limits ferroptosis by blocking DPP4 activity. *Cell Rep.* 20 1692–1704. 10.1016/j.celrep.2017.07.055 28813679

[B47] Xu L. Deng C. Pang B. Zhang X. Liu W. Liao G. (2018). TIP: a web server for resolving tumor immunophenotype profiling. *Cancer Res.* 78 6575–6580. 10.1158/0008-5472.can-18-0689 30154154

[B48] Yan Y. Huang Z. Cai J. Tang P. Zhang F. Tan M. (2020a). Identification of a novel immune microenvironment signature predicting survival and therapeutic options for bladder cancer. *Aging* 12:2327. 10.18632/aging.202327 33408272 PMC7880321

[B49] Yan Y. L. Huang Z. N. Zhu Z. Cui Y. Y. Li M. Q. Huang R. M. (2020b). Downregulation of TET1 promotes bladder cancer cell proliferation and invasion by reducing DNA Hydroxymethylation of AJAP1. *Front. Oncol.* 10:667. 10.3389/fonc.2020.00667 32528872 PMC7253684

[B50] Yang J. Antin P. Berx G. Blanpain C. Brabletz T. Bronner M. (2020a). Guidelines and definitions for research on epithelial-mesenchymal transition. *Nat. Rev. Mol. Cell. Biol.* 21 341–352. 10.1038/s41580-020-0237-9 32300252 PMC7250738

[B51] Yang Y. Luo M. Zhang K. Zhang J. Gao T. Connell D. O. (2020b). Nedd4 ubiquitylates VDAC2/3 to suppress erastin-induced ferroptosis in melanoma. *Nat. Commun.* 11:433. 10.1038/s41467-020-14324-x 31974380 PMC6978386

[B52] Yu F. Quan F. Xu J. Zhang Y. Xie Y. Zhang J. (2019). Breast cancer prognosis signature: linking risk stratification to disease subtypes. *Brief Bioinform.* 20 2130–2140. 10.1093/bib/bby073 30184043

[B53] Yuan H. Li X. Zhang X. Kang R. Tang D. (2016). CISD1 inhibits ferroptosis by protection against mitochondrial lipid peroxidation. *Biochem. Biophys. Res. Commun.* 478 838–844. 10.1016/j.bbrc.2016.08.034 27510639

