# Supplementary material for: A Novel Ferroptosis-Related Prognostic Signature Reveals Macrophage Infiltration and EMT Status in Bladder Cancer
Source: Front Cell Dev Biol. 2021 Aug 20;9:712230. doi: 10.3389/fcell.2021.712230 (PMC8417704; doi:10.3389/fcell.2021.712230)
Supplement: Supplementary file 1 [file Table_1.DOCX]

Supplementary Material


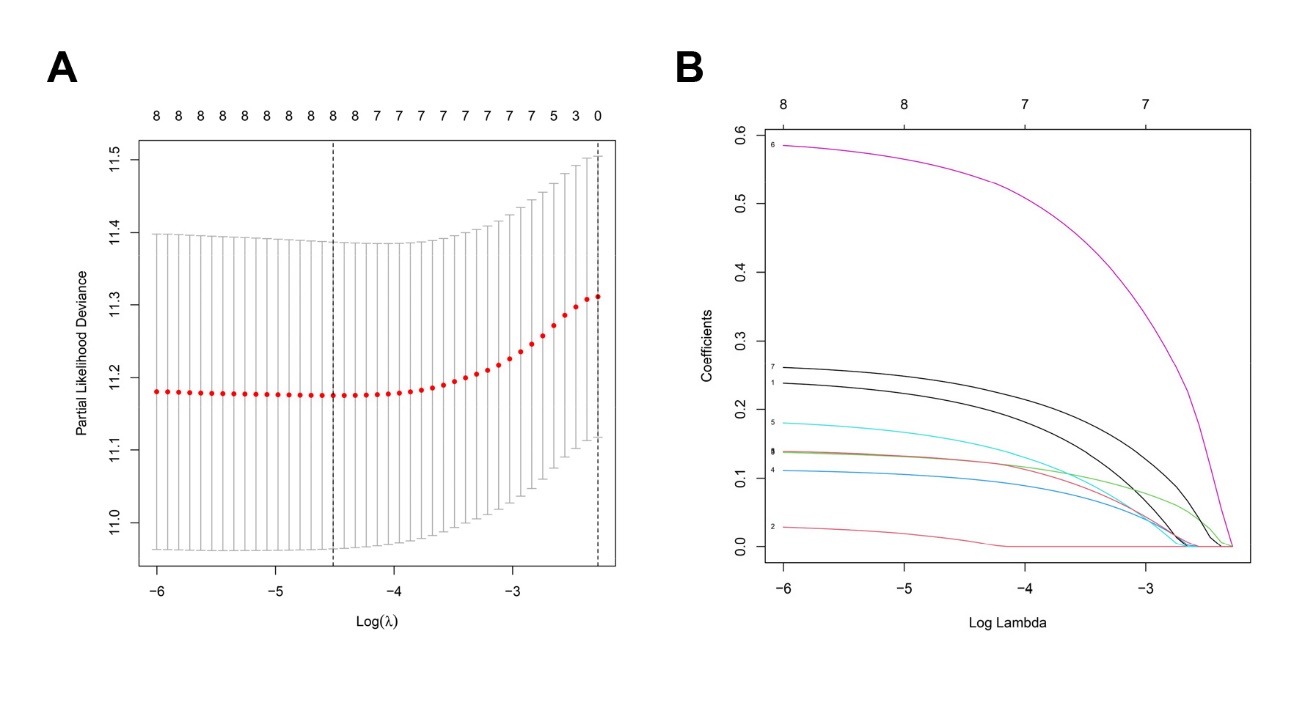


**Supplementary Figure 1.** Lasso regression analysis of ferroptosis-related genes with prognostic potential in the training cohort of BC patients. (**A, B**) Lasso regression analyses of differentially expressed genes using the OS model. The optimal values of the penalty parameter λ were determined by cross validation.


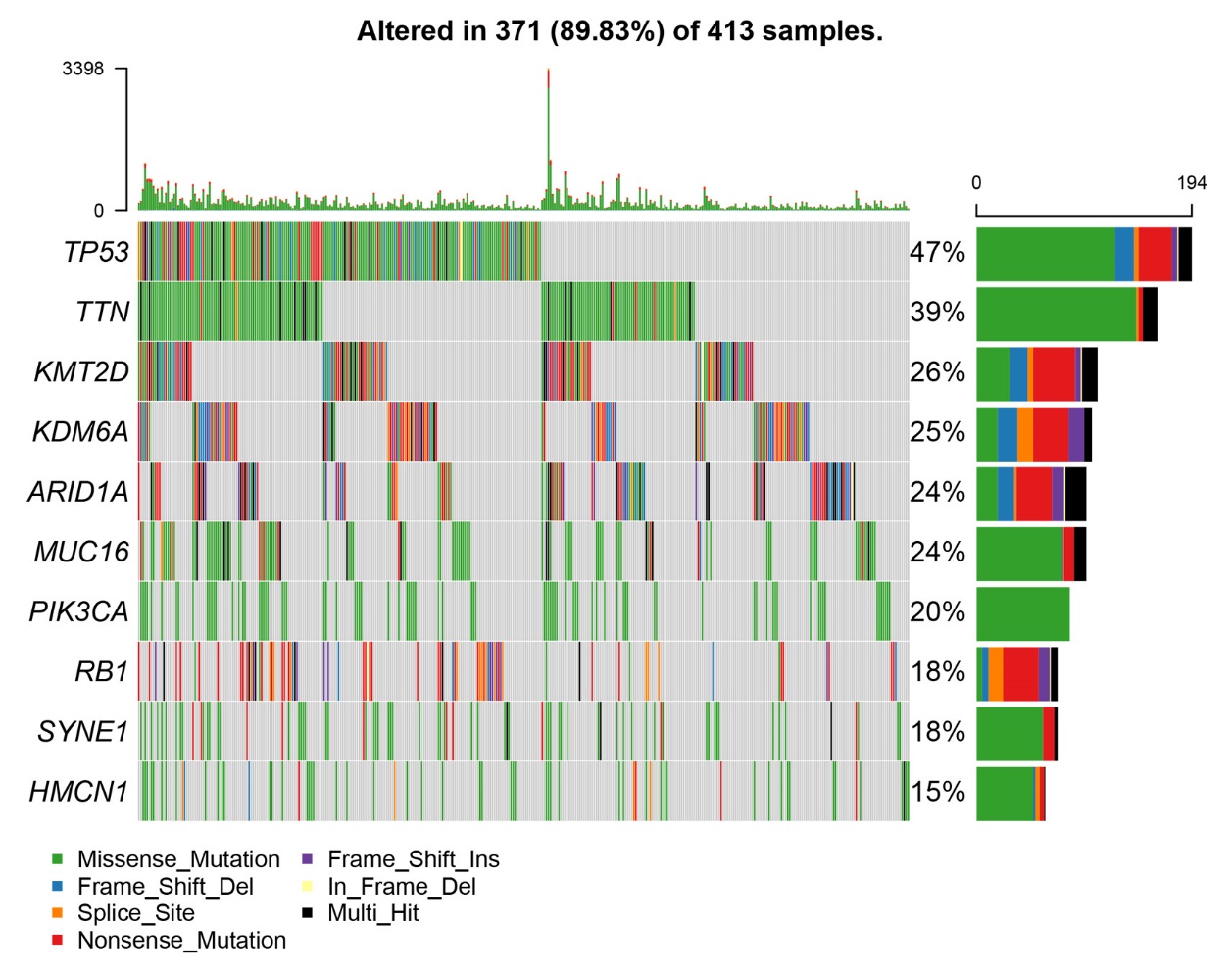


**Supplementary Figure 2.** Landscape of mutation profile in TCGA-BLCA dataset.
